# Draft Genome Sequence of Erwinia psidii, Causal Agent of Bacterial Blight of Guava (*Psidium guava*) and Dieback of Eucalypt (*Eucalyptus* spp.)

**DOI:** 10.1128/MRA.01598-18

**Published:** 2019-01-31

**Authors:** Pollyane da Silva Hermenegildo, Samuel A. Santos, Lúcio M. S. Guimarães, Isadora C. Pereira, Pedro M. P. Vidigal, Jorge L. Badel, Poliane Alfenas-Zerbini, Reginaldo G. Mafia, Marisa A. S. V. Ferreira, Acelino Couto Alfenas

**Affiliations:** a Laboratory of Phytobacteriology, Department of Plant Pathology, Universidade de Brasília, Federal District, Brazil; b Laboratory of Forest Pathology, Department of Plant Pathology, Instituto de Biotecnologia Aplicada a Agropecuária (BIOAGRO), Universidade Federal de Viçosa, Minas Gerais State, Brazil; c Núcleo de Análise de Biomoléculas (NuBioMol), Centro de Ciências Biológicas, Universidade Federal de Viçosa, Minas Gerais State, Brazil; d Laboratory of Molecular Phytobacteriology, Department of Plant Pathology, Universidade Federal de Viçosa, Minas Gerais State, Brazil; e Department of Microbiology, Instituto de Biotecnologia Aplicada a Agropecuária (BIOAGRO), Universidade Federal de Viçosa, Minas Gerais State, Brazil; f Centro de Tecnologia, Fibria Celulose S.A., Aracruz, Espírito Santo State, Brazil; Georgia Institute of Technology

## Abstract

Here, we present a draft genome sequence of the type strain IBSBF 435 of Erwinia psidii (*Enterobacteriaceae*), a phytopathogen that causes bacterial blight on guava (Psidium guava) and dieback and wilt on eucalypt (*Eucalyptus* spp.), both of which are important emerging diseases.

## ANNOUNCEMENT

Erwinia psidii was first described in Brazil in 1983 as causing bacterial blight on guava plants in Pindamonhangaba and Valinhos in the Brazilian state of São Paulo (1). The pathogen has spread to the Brazilian states of Minas Gerais (2), Espírito Santo (3), Distrito Federal (4), and Paraná (5). The disease has been shown to be of great importance since it causes significant losses in guava fruit production (6). Although the disease is currently restricted to Brazil, it is considered a potential risk for other guava-producing countries, such as India, Egypt, Mexico, and Pakistan (7). Recently, E. psidii was also reported to cause dieback and wilt in eucalypt plantations in Argentina, Uruguay, and Brazil, and polyphasic taxonomy in association with multilocus sequence analysis was used to confirm the taxonomy of this patoghen (8, 9). In Brazil, the disease on eucalypt has been observed in the states of Rio Grande do Sul, São Paulo, and Mato Grosso do Sul (9). Disease incidence may reach up to 100% in some areas and seasons, greatly reducing the productive capacity of the eucalypt crop (9).

In both hosts, the main symptoms caused by the pathogen consist of petiole necrosis and tanning of the central leaf vein. Macroscopic and microscopic ooze may emerge from the lesions. Necrosis and mummification of flowers and young fruits are often observed in guava, whereas wilt is observed in eucalypt trees, which may culminate with their death in the field (9, 10). In recent years, studies have been focused on pathogen detection (10, 11), host colonization (5, 12), and genetic variability in the pathogen populations (13). However, the genetic basis of E. psidii virulence remains unclear. Thus, the aims of this study were to sequence and annotate the whole genome of the type strain of E. psidii IBSBF 435 (also known as ATCC 49406, CFBP 3627, CIP 105200, DSM 17597, ICMP 8426, LMG 7039, NCPPB 3555, and PDDCC 8426), isolated from guava plants (1).

The IBSBF 435 strain was obtained from the Phytobacteria Culture Collection of Instituto Biológico in Campinas, São Paulo State, Brazil. The strain was retrieved from a stock in 30% glycerol at −80ºC and grown in petri plates containing solid 523 medium (14) at 28°C for 48 hours. Then, the bacterial cells were used for DNA extraction using a Wizard genomic DNA purification kit (Promega, Madison, WI, USA), following the manufacturer’s instructions. For whole-genome sequencing of E. psidii, libraries were prepared from bead size-selected DNA fragments from DNA samples and sequenced using the Illumina NovaSeq 6000 platform with 2 × 150-bp paired-end reads, according to the manufacturer’s instructions. A high sequencing coverage of about 200-fold was obtained. Low-quality reads were trimmed and filtered by using AfterQC with default parameters selected (15). High-quality reads were *de novo* assembled into scaffolds using SPAdes version 3.10.1 with testing of different k-mers (from 23 to 123) (16). Gene prediction and annotation were performed by using the NCBI Prokaryotic Genome Annotation Pipeline (PGAP). The completeness of the assembled genome was estimated using Benchmarking Universal Single-Copy Orthologs (BUSCO) (17).

The size of the assembled E. psidii IBSBF 435 genome is 4.5 Mb, and its G+C content is 51.3%. The assembly contains 42 scaffolds with sizes ranging from 1,000 nucleotides (nt) to 628 kb, an average scaffold length of 107 kb, an *N*_50_ value of 268 kb, and a total of 3,954 predicted genes. The assembly had an average coverage of 60-fold. The completeness assessment of the genome by BUSCO identified 97.5% complete and single-copy, 2.5% missed, and no duplicated or fragmented gene groups specific for bacteria, which indicates that the assembled genome covers most of the coding regions. The quality of our assembly is comparable to that of strains belonging to other *Erwinia* species, such as E. mallotivora (18), E. tracheiphila (19), and E. amylovora (20). This is the first draft genome sequence of the plant-pathogenic bacterium E. psidii, which may be used as a reference for the species. This genome sequence will be an invaluable molecular tool for gaining a better understanding of the biology of the interaction of this important emerging pathogen with its host plants, by using diverse scientific approaches, including comparative genomics.

### Data availability.

The sequences from this whole-genome shotgun project were deposited in GenBank under accession number RHHM00000000. The raw data were also deposited in the Sequence Read Archive (BioProject number PRJNA498492).
